# METABOLIC CONSEQUENCES OF METHIONINE REDOX IN METHIONINE RESTRICTION

**DOI:** 10.1093/geroni/igz038.398

**Published:** 2019-11-08

**Authors:** Kevin Thyne, Yuhong Liu, Adam B Salmon

**Affiliations:** 1 University of Texas, Health Science Center at San Antonio, San Antonio, Texas, United States; 2 University of Texas Health at San Antonio, San Antonio, Texas, United States

## Abstract

While caloric restriction (CR) provides highly robust improvements to longevity and health, dietary restriction of the essential amino acid methionine can provide similar benefits including improved metabolic function and increased longevity. Despite these similarities between CR and methionine restriction (MR), there is growing evidence to suggest they may be mediated by different mechanisms that require further elucidation. The sulfur side-chain of methionine is highly prone to oxidation, even in vivo, with redox changes of these residues potentially altering protein function and interfering with its use as a substrate. An entire family of enzymes, methionine sulfoxide reductases, have evolved in aerobic organisms to regulate the redox status of methionine. We tested the role of methionine sulfoxide reductase A (MsrA) in the physiological and metabolic benefits of MR. After three months of MR, mice lacking MsrA (MsrA KO) showed significant loss of weight, including both fat and lean mass, in comparison to wild-type mice under MR. Both MsrA KO and wild-type mice responded to MR with improvements to both glucose and insulin tolerance. However, MR MsrA KO mice showed lower HbA1c and reduced leptin compared to MR wild-type mice. Overall, our results show mice lacking MsrA have a stronger response to MR suggesting that methionine redox may play an important role in some of the mechanisms responsible for these metabolic outcomes. Further studies clarify whether MsrA could also be a potential regulator of the longevity benefits of MR.

